# Supplementary material for: The risk of bias in denoising methods: Examples from neuroimaging
Source: PLoS One. 2022 Jul 1;17(7):e0270895. doi: 10.1371/journal.pone.0270895 (PMC9249232; doi:10.1371/journal.pone.0270895)
Supplement: Attachment — Submitted filename: KayManuscriptResponseToReviewers.docx [file pone.0270895.s001.docx]

**Response to Reviewer 1**

**Point 1.1.** “The author highlights the bias introduced by denoising and that lower MSE with respect to the ground truth does not always mean lower bias. Overall, the manuscript was well written with comprehensive demonstrations of the bias after denoising with different methods.”

We thank the reviewer for their time and effort in reviewing this manuscript. We appreciate the reviewer’s suggestions, and we have revised the manuscript to accommodate the suggestions (details below).

**Point 1.2.** “My main concerns are 1. Is bias bad? Unbiased estimator does not always exist and sometimes biased estimator can lower MSE [1,2,3] (similar to Fig. 1, top right). While Fig.3 clearly indicates bias changes the shapes of the estimated HRF, Fig. 2 is not as clear. Gaussian smoothing introduced larger bias compared to anisotropic smoothing but is it bad and how it would affect, for example, clinical diagnosis? Except from the fact the Gaussian smoothing images are blurry (which is uncorrelated to bias), it is still unclear how the bias is dangerous in this case. Perhaps showing an example of bias can obscure a structure of interest (tumor) would highlight the point.”

Here, the reviewer highlights interesting and valuable perspectives on the issue of bias. We agree with the reviewer that: (i) biased estimators can lower MSE and (ii) bias is not necessarily a problem in all situations (e.g., if an image is a little blurry, this might not be a big deal to a clinician who is interpreting the image).

We appreciate the suggestion that one could construct examples where a denoising method might obscure a structure of interest (e.g. tumor). We feel that this would not quite fit the tone/style of the current manuscript (and might actually convey the wrong impression unless substantial work was done to contextualize that observation). But we do think this is, in general, a really good idea and might form the basis of a separate project where specific realistic state-of-the-art denoising methods are shown to potentially result in major problems of bias!

**Action:** To clarify the issues raised by the reviewer, the revised manuscript now explicitly discusses the idea that, often, bias is intentionally introduced to an estimator in order to reduce MSE (see p. 11, lines 278–280; p. 21, lines 535–536). Also, the underlying issue here might be that the manuscript seems too strongly against the idea of bias. In order to help balance the discussion and accommodate the reviewer’s point, the revised manuscript now includes additional text that acknowledges that bias is sometimes okay (see p. 22, lines 575–577). Finally, we thank the reviewer for the literature suggestions; we have reviewed these suggestions, and cite the suggestions in the revised manuscript (see p. 21, lines 535–536).

**Point 1.3.** “2. How bias affect downstream analyses? Continuing point 1 above, some subsequent analyses should be done to highlight how bias would affect the results and how severe it could be. In the context of MRI in this paper, it could be segmentation from sMRI, correlation between stimuli and signal from fMRI, or tensor fitting and tractography from dMRI. Without subsequent analyses, although we acknowledge there is a bias, it is still unclear whether we should spend effort removing it with the cost of increasing variance (e.g., using parametric fit instead of basis restriction in Fig.3).”

Here, the reviewer raises the point that bias, in and of itself, is not the “end result”, and that what ultimately matters to the practitioner is the downstream impact of bias. We agree with the reviewer. However, we would like to point out that there are many diverse ways to use a set of data, and the examples demonstrated in this paper are very diverse in and of themselves. Thus, it is challenging to think about how one might show some examples without complicating the paper too much and without making the examples too narrow-scoped.

**Action:** In line with the helpful suggestion from the reviewer, we have performed new analyses showing the downstream impact of the denoising methods in the T1 anatomy example (Figure 2) on tissue segmentation (see p. 17–18). The results provide interesting insights into the practical consequences of bias. We also revise the manuscript to include some additional discussion (as suggested by the reviewer) about how taking steps to remove bias might incur other complications (see p. 22, lines 584–586).

**Point 1.4.** “3. Is MSE still a good performance measurement? It is obvious that smaller bias and variance are favored. But in case of a trade-off between variance and bias like many methods shown in this work, is measuring bias alone a good practice? MSE accounts for both bias and variance and is a good measure to balance out the two terms, especially when to determine in a decrease in bias/variance worth it (given an increase in the other one). Again, this highlights the importance of demonstrating how bias would affect the images visually and subsequent analyses. If it does not affect those significantly, it might be sufficient to quantify MSE alone.”

Here, the reviewer points out that MSE has many appealing features as a metric, and that measuring bias alone might not be a good idea.

We agree with the reviewer. We did not mean to imply that MSE is not valuable. We also did not mean to imply that we should only think about bias.

**Action:** We have revised the manuscript to better clarify the discussion of MSE, bias, and variance (see p. 20, line 519 through p. 21, line 542). We hope the revised text provides a clearer and more balanced discussion of these issues.

**Point 1.5.** “Minor comment: While the title is "The risk of bias in denoising methods", 2 out of 3 experiments are MRI-related. The title and the abstract should make clear that the manuscript experiments are limited to MRI data.”

**Action:** We acknowledge this suggestion. We have revised the title of the manuscript accordingly (see p. 1, line 3).

**Point 1.6.** “Although experiments are meant to demonstrate how bias would be introduced, the paper is undermined that little to no state-of-the-art denoising method was used (while they are mentioned and discussed many times throughout the manuscript).”

We agree with the reviewer that one limitation of the current manuscript that it does not specifically implement state-of-the-art denoising methods. As we mention in the manuscript (see p. 12, line 296), this is a deliberate choice on our part, as we are attempting to construct very clear and general examples that demonstrate general principles. However, we agree that it would be possible to construct a very different type of paper that addresses specific methods proposed in the literature.

**Response to Reviewer 2**

**Point 2.1.** “Reviewer #2: This work studies the risk of bias in denoising methods. More specially, denoising methods may introduce bias, which has risks leading to incorrect scientific inferences. To investigate the denoising-induced bias, three simulation experiments are performed, which draw three key conclusions and the importance of quantifying the possibly denoising-induced bias. The paper is well written and easy to follow. The conclusions from this work can benefit a large number of denoising works and provide valuable guidance for the use of denoising methods.”

We thank the reviewer for their time and effort in reviewing this manuscript, and appreciate the thoughts and suggestions.

**Point 2.2.** “Regarding this work, I only have several minor concerns. 1. The first simulation is based on Gaussian noise corrupted MR images. However, depending on the k-space data acquisition schemes and reconstruction approaches, the noise in MR images follows a stationary/non-stationary Rician/non-central Chi noise [1,2]. I am wondering whether the Gaussian noise assumption has some influences on the experimental results.”

Here, the reviewer points out that in real MRI data, noise is not simple Gaussian noise, but is better described as Rician and/or has more complicated structure (depending on complex interactions with exactly how the data are acquired).

We agree with the reviewer fully, and we thank the reviewer for the literature suggestions. It is certainly the case that the simulation results are technically valid for only the assumptions that it incorporates. We imagine that, yes, results might vary if one were to explore other types of noise, and that would be certainly interesting to explore. For the purposes of the present manuscript, we are trying to “keep things simple” in order to deliver on our goal of presenting clear, simple concrete simulations that highlight and illustrate the issue of bias.

**Action:** We have reviewed and cited the provided literature suggestions, and we have revised the manuscript to acknowledge and highlight the point that Gaussian noise is certainly a simplification compared to real MRI noise (see p. 12, lines 302–304).

**Point 2.3.** “2. It is also helpful to provide some insightful discussions on how to define a good denoising method, which can benefit the medical image computing community.”

Here, we interpret the reviewer’s comment to mean that it would be helpful if the manuscript provided a clearer and more direct discussion of what we feel is a good denoising method.

**Action:** In line with this useful suggestion, we have revised the discussion in the manuscript and provide some thoughts on this matter (see p. 23, lines 597–608).
